# Performance Analysis of Scattering-Level Multiplexing (SLMux) in Distributed Fiber-Optic Backscatter Reflectometry Physical Sensors

**DOI:** 10.3390/s20092595

**Published:** 2020-05-02

**Authors:** Daniele Tosi, Carlo Molardi, Wilfried Blanc, Tiago Paixão, Paulo Antunes, Carlos Marques

**Affiliations:** 1 School of Engineering and Digital Sciences, Nazarbayev University, Nur-Sultan 010000, Kazakhstan; carlo.molardi@nu.edu.kz; 2 Laboratory of Biosensors and Bioinstruments, National Laboratory Astana, Nur-Sultan 010000, Kazakhstan; 3 INPHYNI-CNRS UMR 7010, Université Côte d’Azur, Parc Valrose, 06108 Nice, France; wilfried.blanc@inphyni.cnrs.fr; 4 Physics Department, I3N & University of Aveiro, 3810-193 Aveiro, Portugal; tiagopaixao@ua.pt (T.P.); pantunes@ua.pt (P.A.); carlos.marques@ua.pt (C.M.)

**Keywords:** optical fiber sensors, optical backscatter reflectometry (OBR), distributed sensors, scattering-level multiplexing, Rayleigh scattering

## Abstract

Optical backscatter reflectometry (OBR) is a method for the interrogation of Rayleigh scattering occurring in each section of an optical fiber, resulting in a single-fiber-distributed sensor with sub-millimeter spatial resolution. The use of high-scattering fibers, doped with MgO-based nanoparticles in the core section, provides a scattering increase which can overcome 40 dB. Using a configuration-labeled Scattering-Level Multiplexing (SLMux), we can arrange a network of high-scattering fibers to perform a simultaneous scan of multiple fiber sections, therefore extending the OBR method from a single fiber to multiple fibers. In this work, we analyze the performance and boundary limits of SLMux, drawing the limits of detection of N-channel SLMux, and evaluating the performance of scattering-enhancement methods in optical fibers.

## 1. Introduction

Optical fiber sensors have been consolidated in the past decades, and they are now an established technology in several applicative fields [1,2,3,4]. The key advantage of optical fiber sensors, with respect to other sensing technologies, such as piezoelectric, microelectromechanical systems (MEMS), or other mechanical or electronic sensors, is the possibility of interrogating multiple sensors placed upon a single fiber [2,3]. In this case, a single optical fiber sensing system can host several sensors, and therefore it is possible to perform a simultaneous detection of hundreds [2], thousands [5], or even up to a million sensing points [6]. This possibility outperforms wireless sensor networks in terms of sensing distribution [3].

Multiplexing is the key to access advanced sensing applications, and it represents the current frontier of fiber optic sensing. By multiplexing, we define the act of allocating a single optical fiber cable to a plurality of sensors, and disambiguating their detection by means of a “diversity” feature. Each sensor must be orthogonal, or quasi-orthogonal, to the other sensors, making it possible to simultaneously detect a plurality of sensing data, and then isolate the contribution of each sensor. In this sense, the definition of multiplexing in sensor networks is similar to its implementation in telecommunications, and research trends often intersect in these two areas [1].

Time-division multiplexing (TDM) and wavelength-division multiplexing (WDM) represent the golden standard of multiplexing in optical fiber sensors, as they apply to Fiber Bragg Grating (FBG) sensors [1,2]. The FBG is the most popular optical fiber sensor, as it can be inscribed virtually on any fiber [7], and FBG arrays can be easily fabricated, with spacing ranging from several kilometers down to the millimeter [8]. TDM is implemented through an optical switch (1 × T), which commutes to select a single channel from a network of T channels. WDM is implemented by inscribing FBGs at different wavelengths, dividing the bandwidth of an interrogator into W individual slots. TDM and WDM are used by modern FBG interrogators, which can interrogate a network up to T × W FBG sensors [2,8]; for example, an 8-channel interrogator with a 100 nm bandwidth can interrogate up to 400 FBGs spaced by 2 nm, which is a “safe” spacing between adjacent wavelength elements.

Building on this result, recently, several advanced multiplexing techniques have been presented and applied to fiber optic sensors, exploiting different degrees of diversity. Gasulla et al. [9] presented a spatial division multiplexing (SDM) method based on a multi-core fiber, where in this work, the diversity is given by seven well-spaced fiber cores, each hosting a set of sensors, considering that the separation between the cores does not lead to modal interference. The two fiber polarizations have been exploited by Oh et al., using FBG sensors [10]; this configuration, based on polarization-division multiplexing (PDM), exploits the different sensitivity of the fast/slow-axis polarized light in an FBG to interrogate a single FBG for both strain and temperature. Recently, multiplexing has been extended to Fabry–Perot sensors in the cepstrum domain [11], and even to smartphone-based optical sensors using an SDM method applied to the phone camera [12].

Distributed optical fiber sensors represent the main alternative to the network of multiplexed sensors [5]. Distributed sensors interrogate the multiple reflections, due to scattering events, occurring in an optical fiber cable. Optical backscatter reflectometry (OBR) is one of the most important methods, and as demonstrated first by Froggatt et al. [13], OBR interrogates the Rayleigh spectral signals, usually labeled as “signatures” of the fiber in each location, with a theoretical resolution of 10 μm. OBR has found initial applications in the monitoring of optical systems [14], and has been subsequently extended to sensors, with particular applications in biomedical engineering [15].

OBR is inherently a single-fiber system, whereas a fiber span connected to the OBR is interrogated; TDM is possible by using an optical switch that selects multiple fibers, but that process hampers the rapid detection of signals due to the need to acquire multiple triggers [16,17]. Since the OBR method has a spatial resolution, it is possible to arbitrarily displace the fiber in order to detect strain or temperature in multiple arrangements; for example, Macchi et al. [15] reported a planar temperature detection with a fiber disposed along eight radii during an ex-vivo radiofrequency ablation, while Parent et al. [17] reported a shape sensing method based upon a fiber triplet.

**Figure 1:**
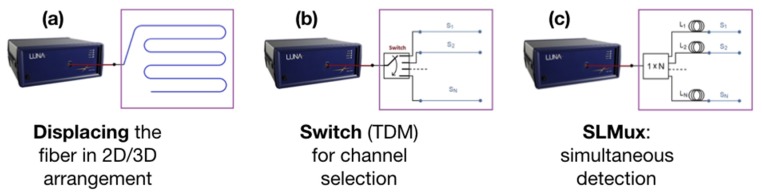
Schematic of the optical backscatter reflectometry (OBR) setup for the measurement of planar (2D) or tridimensional (3D) arrangements. (**a**) The fiber is displaced in a 2D/3D configuration, as in [15]; this is impractical for several medical devices, due to limited spacing, tight bending and excessive mechanical torsions of the fiber. (**b**) The alternative is to use a 1 × N switch (TDM arrangement), to single out each individual channel [17,22]; in this arrangement, the OBR loses a large portion of real-time sensing, due to the significant increase of measurement time (N-fold increase, with ideal switches and optimal software), well over 1 s. (**c**) The SLMux setup [18,19], presented in this work; the switch is substituted by a splitter, hence the detection is simultaneous and real-time (0.3 s). A network of single-mode fiber delayers and high-scattering fiber multiplex from a single fiber to the N-size sensing network, each constituted by a distributed sensor.

However, in some applications, particularly involving medical applications which require a high-density sensing in planar or tridimensional structures [4], it is simply not possible, or not convenient, to use a single fiber, and multiple fibers are needed. To circumvent this stalemate, Beisenova et al. recently pioneered a configuration labeled scattering-level multiplexing (SLMux) [18,19], which makes use of high-scattering fibers characterized by MgO-based nanoparticle doping in the fiber core [20]. This architecture uses, as a diversity element, the amount of scattered power in each location, and can interrogate multiple locations on different fibers. This approach has been, so far, reported for the measurement of strain [18] and shape [21] on a medical needle, and for a planar temperature measurement in a mini-invasive thermotherapy [19]. This scenario is sketched in Figure 1, while OBR has been presently used with fibers arranged in an arbitrary shape, or using a switch to multiplex multiple fibers in time [17,22], SLMux is the only method that allows a simultaneous scan of multiple sensing fibers.

In this work, we provide for the first time a quantitative evaluation of the performance of SLMux, taking into account the multiple factors that affect performance. Our analysis is based on the evaluation of all factors that affect the propagation of light into high-scattering fibers, evaluating the key performance indicators, and thus the maximum lengths of those fibers used for sensing.

The paper is arranged as follows: Section 2 describes the principle of the operation of SLMux, and of the MgO-based nanoparticle-doped fibers that implement it; Section 3 provides a model for the implementation and analysis of SLMux based on fiber performance, and draws the performance parameters; Section 4 evaluates the performance boundaries of the SLMux, and presents the main findings; Section 5 analyzes a range-extension to high-scattering fibers using FBGs; Section 6 discusses the results of the performance analysis in the light of the biomedical applications; finally, Section 7 draws the conclusions.

## 2. Scattering Level Multiplexing: Method and Implementation

### 2.1. Principle of Operation

**Figure 2:**
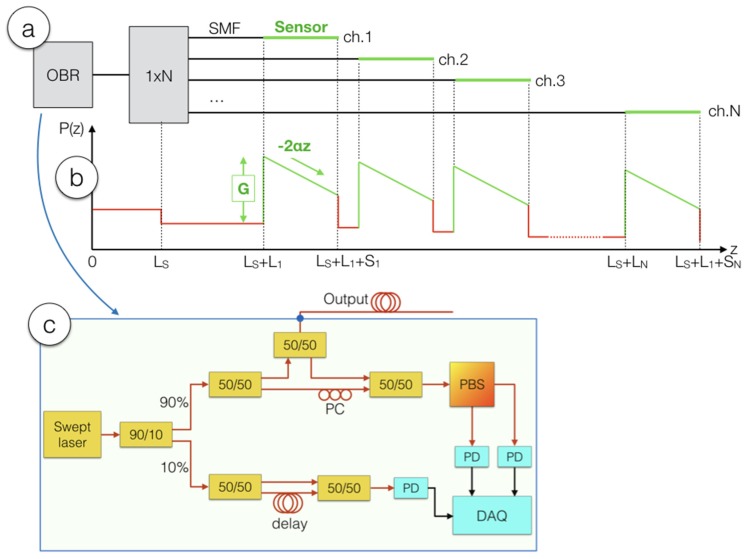
Principle of operation of the Scattering-Level Multiplexing (SLMux) setup. (**a**) Experimental setup implementing the SLMux, in the N-channel system; (**b**) power scattering trace, reporting the power P at each location z measured on the OBR in the SLMux setup; (**c**) detail of the swept-laser interferometer included in the OBR instrument (PD: photodetector, PBS: polarization beam splitter).

The principle of operation of the SLMux is sketched in Figure 2, and is designed to multiplex an OBR instrument into multiple sensing regions, based on the scattering amount and length of fibers [19]. The OBR source used in our experiments, and for which all scattering power levels are referenced, is the Luna OBR4600 (Luna Inc., Roanoke, VA, US), which is the most common implementation of OBR. The instrument, used in Figure 2a as the light source and detector, is sketched in Figure 2c. The OBR is a swept laser interferometer which has a measurement arm (the upper circuitry) and a trigger that serves as a delay line [13,16].

The OBR is connected to a 1 × N splitter, to *N* separate channels. A network of single-mode fibers (SMFs), each having length *L_i_*, *i* = 1,…, *N*, is used to separate the sensing fibers. The sensing fibers are required to have a higher scattering than the SMF, in order to make the system operate without interference, and they are connected to the tail of the SMF by means of splicing. Within the high-scattering sensing fibers, the OBR works as a distributed sensor, with spatial resolution corresponding to *c*/(2 *n_eff_* Δ*f*) [5], *c* = speed of light, *n_eff_* = effective refractive index of the fiber, Δ*f* = the frequency range of the swept laser. The length of the i-th sensing fiber is *S_i_*.

The scattering trace *P*(*z*), *z* = fiber length, *P* = backscattered power, is shown in Figure 2b in dB units, and appears different from the standard OBR traces. Unlike the standard OBR arrangements of Figure 1a,b, the SLMux system is not engineered to work on the whole fiber chain, but only on the high-scattering fibers. The implication, looking at the scattering trace, is that the distributed sensing works only on the green portions of the trace of Figure 2b, which correspond to the specific sensing fibers, and does not work on the SMF chain, in the red portions of the trace. The chart explains the function of the network of SMF fibers, here acting as delay lines or spacers to shift the position of each sensing fiber at a different length along *z*.

The scattering trace has a sawtooth shape [18]; when moving from the SMF to a high-scattering fiber, the increased amount of scattering provides an increment of signal on the OBR, which we define as the scattering gain (*G*). On the other hand, given that Rayleigh scattering is much stronger in these fiber elements, the losses are much higher, and they appear as a linear decrease with −2α*z* with the two-way fiber attenuation (2α).

Signal demodulation is performed as described in [16]. Cross-correlation between the Rayleigh signatures (i.e., the spectral reflectivity values acquired at each location), acquired in reference and measurement conditions, are calculated for each point along *z* [4], selecting a window that comprises all of the sensing fibers. Then, the individual contribution of each sensor is isolated, since the scattering at the location is larger than the surrounding fiber.

### 2.2. High Scattering Fibers

Rayleigh scattering is known to be a primary factor for losses in optical fibers, and therefore SMF fibers are engineered to minimize its contribution in order to reduce the fiber attenuation, which is necessary for long-haul communications. The latest research carried out in OBR-distributed sensing, however, has almost the opposite trend, namely, developing new methods to enhance scattering events in an optical fiber, in order to have a strong scattering gain. Loranger et al. [23] presented a method based on the UV exposure of a single-mode fiber, obtaining a scattering enhancement of approximately 20 dB. Yan et al. [24] showed a scattering enhancement of 40 dB by inscribing a nanograting in the fiber through a femtosecond laser.

The solution proposed by the authors, instead, makes use of a fiber doped with nanoparticles based on MgO (MgO-NP) within the core as the sensing fiber, which guarantees a scattering gain up to 49 dB. A detail of the fabrication of the fiber was previously reported by Blanc et al. [20,25] and subsequently by Beisenova et al. [18,19,21], while Section 4 of this work describes the specific scattering performance of the fiber in the context of SLMux.

In short, the MgO-NP fiber is designed to have the same core and cladding size of the SMF (10/125 μm inner/outer diameter), while having a higher density of scattering sources in the proximity of the fiber core, giving rise to a much higher Rayleigh scattering. Fibers have been drawn from silica preforms made with modified chemical vapor deposition (MCVD) [25]. The addition of magnesium during the fabrication activates the formation of Mg-silicate nanoparticles [26], as reported by Blanc et al. [20], which act as a scattering source for input signals.

The MgO-NP method to enhance Rayleigh scattering is the most interesting from the application standpoint, because it leads to the fabrication of a proper optical fiber, rather than exposing a portion of a pre-existing fiber. The MgO-NP can be spooled like a normal fiber, and can be spliced in a standard fusion splicer; in all of the following characterization, MgO-NP fibers have been spliced to standard SMF in a low-cost splicer (Fujikura 12S, SMF-SMF recipe). Since the fiber can be spooled, spliced and treated as a normal fiber, it also does not require removing the protective jacket around the fiber, as instead done in previous works [22,23,24]. The fiber used in experiments, having a standard 250 μm jacket, is much more suitable for working on medical devices such as epidural (Tuohy) [21] or Chiba [18] needles, without any recoating that would increase the fiber thickness.

## 3. Theoretical Analysis of Scattering-Level Multiplexing

### 3.1. Definitions

Table 1 shows the parameters used in the following theoretical analysis. For simplicity, all power values are reported in dBm units, all attenuation and gains in dB, and fiber attenuation in dB/m. In calculations, attenuations and losses are always referred as two-way, i.e., accounting both the forward and backward propagation. Here, gain terms refer to the increment of the scattering level with respect to standard SMF fibers, and they do not imply an amplification of optical power.

### 3.2. Scattering Diversity and Power Propagation

The key principle of SLMux is avoiding the overlap between two sensing regions, at any given space; in this case, we can imply that the power backscattered by the MgO-NP sensor is much larger than the combination of the other SMF fibers overlapping in the same location, since the scattering gain is large. We can label this condition as the “scattering diversity”, the underlying process of SLMux. In formula, as derived from Figure 2:

(1) $$\left[\left(L_{S}+L_{i}\right)÷\left(L_{S}+L_{i}+S_{i}\right)\right]\;\neq \;\left[\left(L_{S}+L_{j}\right)÷\left(L_{S}+L_{j}+S_{j}\right)\right]$$

for any pair (*i*, *j*) = 1,…, N. Here the diversity symbol (≠) refers to the entire range of extension of the *i*-th and *j*-th sensing regions, which have to avoid any overlap.

With this parameter layout, and considering that the OBR works only in the sensing regions corresponding to high-scattering fibers, for each *i*-th channel, we can then express the backscattered power, in ideal conditions, as:

(2) $$P\left(z\right)=P_{SMF}-10\log_{10}N+G-2\alpha \left(z-L_{S}-L_{i}\right).$$

for from the location (*L_S_* + *L_i_*) ≤ *z* ≤ (*L_S_* + *L_i_* + *S_i_*). The power follows the sawtooth shape shown in Figure 2: the baseline power scattered by the SMF (*P_SMF_*) suffers the attenuation term 10log_10_*N* that takes into account the optical splitter, then it is enhanced by the MgO-NP, and decreases linearly following the fiber attenuation.

### 3.3. Underlying Considerations

In order to analyze the SLMux performance, we provide the following considerations, that are mostly verified experimentally. Several of these statements have been previously presented by Beisenova et al. [19]; The appendices contain specific elements and experiments aimed at the verification of the most critical assumptions.

1. The SMF fibers have a constant backscattered power *P_SMF_*, and are lossless. Typical attenuation values for SMF fibers (e.g., Corning SMF-28) are around 0.36–0.48 dB/km two-ways, hence the attenuation on a short span of few meters is negligible.
2. The noise power corresponds to the average power due to the electrical and optical noise at the photodetector of the OBR. This value, labeled *P_N_*, is measured in dark conditions, when no fiber is connected to the OBR [16].
3. Since the scattering level from a MgO-NP fiber is high, we operate the OBR with no amplification (e.g., 0 dB electrical amplification set on the OBR instrument). This is necessary to avoid the nonlinearities in the scattering peaks.
4. With these conditions, we can measure *P_SMF_* = −102.7 dBm, and *P_N_* = −110.7 dBm for the OBR instrument used in experiments. The power is here expressed in absolute units, which depend on the input power launched by the OBR laser source. Noise and interference are always calculated as differential terms, hence the results are independent on the power launched by the OBR.
5. We neglect the reflective effect of connectors, which induce a reflective spike on the OBR. That is because connectors are located at SMF-SMF junctions which are not overlapping to any of the sensing fiber, and hence are irrelevant. Connector losses are treated as impairments.
6. Since commercial splitters are mainly 1 × 2^x^, we consider the operative cases of 1 × 2, 1 × 4, 1 × 8, 1 × 16, 1 × 32 and 1 × 64 splitters. We assume the splitter to have an insertion loss of 10log_10_(*N*) in dB, while the excess loss is treated as an impairment. We assume the length of the splitter to be equal for all channels; adjusting the length of each SMF span it is possible to satisfy the scattering diversity outlined in Equation (1). In this case, the performances of the system are scaled to the upper number of channels, e.g., a 1 × 12 system will have the same performance of a 1 × 16.
7. MgO-NP fibers have a gain scattering *G* (defined as the increment of scattered power with respect to the SMF fiber), and two-way attenuation 2*α*. Then *G* and *α* are assumed to be constant on the whole network. Although different portions of the MgO-NP might have uneven scattering performance, in general these values tend to be similar on fibers drawn from the same process, hence we can simply account for the local variability of *G* and *α* as an impairment.
8. MgO-NPs are spliced to the SMF matching the mode profile, in a quasi-lossless splice. This way, we can treat the splice loss as an impairment, but without alteration of the scattering signatures. Splice losses have always been estimated as < 0.1 dB per splice for any MgO-NP.
9. The scattering signature, i.e., the spectral response of the Rayleigh scattering back-reflection evaluated at each location *z*, is a random signal. We approximate the scattering signature of MgO-NP fibers, like SMF fibers, as a random signal having mean power equal to *P*(*z*). Although the signals are not completely flat, their profile is similar to a white noise (see Appendix A).
10. Scattering signatures from different sections of fibers are statistically independent of each other [19] (see Appendix A).
11. For simplicity, we assume all sensing lengths of the MgO-NP fibers have equal value *S*. In most applications [4], sensors have equal lengths, since they are often mounted on a medical device having a defined length.

### 3.4. Noise and Interference Contribution

We introduce two quality factors for the SLMux, which define its performance across the whole sensing network. The first condition relates to the signal-to-noise ratio (SNR), and measures the amount of signal, i.e., power scattered by the fiber, over the noise level of the OBR; the SNR condition exists for any OBR system, and is defined as *SNR*(*z*) = *P*(*z*) − *P_N_*. Unfolding the power propagation law, since the MgO-NP fiber suffers from progressive losses, the worst-case scenario occurs at the far end of the longest sensing channel, which has the weakest overall signal power. Since this is the worst-case scenario, we can then define the network SNR as:

(3) $$SNR_{network}=P_{SMF}-10\log_{10}N+G-2\alpha S-P_{N}$$

where “network” refers to the SNR related to the simple optical sensing network, as laid out in Figure 2, without the contribution of an additional excess loss of components and power unbalances, that will be treated in the next section.

The second quality factor is specific of the SLMux configuration, and relates to the signal over interference ratio (SIR). We define the SIR at the generic location *z* as *SIR*(*z*) = *P*(*z*) − *P_INT_*, i.e., as the ratio between the signal power *P*(*z*) and interference power *P_INT_* due to the presence of several SMF fiber signatures overlapping to the main signature coming from the MgO-NP fiber. As visually expressed in Figure 2, the *i*-th sensing fiber interferes with (*i* − 1) SMF fibers, each carrying a contribution equal to *P_SMF_*. As by assumptions 9–10, multiple overlapping signatures are statistically independent, hence we combine their average power. With these considerations, the lowest value of network SIR can be derived for the shortest fiber, which has (N−1) interferents:

(4) $$SIR_{network}=-10\log_{10}\left(N-1\right)+G-2\alpha S.$$

Comparing Equation (4) with Equation (3), and approximating log_10_ (*N*−1) ≈ log_10_ *N*, we notice that the system, at least in ideal conditions, is dominated by the SIR; in fact, since the OBR is designed in order to have *P_SMF_* well larger than *P_N_* (8.0 dB higher as by measured in assumption 4), we obtain that the SIR is significantly lower than the SNR, and therefore the interference is the limiting factor on SLMux performance. This consideration however can be mitigated when looking at the non-idealities of the system.

### 3.5. Effect of Impairments

SNR and SIR undergo different typologies of impairments, which account for the non-idealities of the system. The SNR is affected by all the losses in addition to the circuit losses, such as excess loss of splitter, connector and splice losses, and the additional attenuations that compose the power budget. The additional losses reduce the signal power, consequentially lowering the SNR by the same amount; we can therefore write the real SNR as:

(5) $$SNR=SNR_{network}-A=P_{SMF}-10\log_{10}N+G-2\alpha S-P_{N}-A$$

where *A* is the contribution of the excess losses. Even in a well-designed SLMux, excess losses of connectors, fibers, splitter and splices can account for several dB, particularly considering that losses are experienced both by forward and backward waves. For example, the design reported by Beisenova et al., which introduces an additional splitter, shows additional excess losses [18,19].

The SIR is not vulnerable to most of the loss terms, since the attenuations affect in the same way the MgO-NP fiber, and the SMF fibers overlapping to it. Instead, the SIR is vulnerable to the loss imbalances, i.e., the deviation of the excess of attenuations or to the local variations of scattering properties from their baseline values. For example, if the splitter has the same excess and connector loss on each channel, both signal and interference terms suffer the same attenuation and their ratio would remain the same. The attenuation of MgO-NP fibers is in general repeatable over fiber samples drawn from the same preform, so the attenuation deviations are also treated as impairments. The parameter that impairs the SIR is the maximum change of the attenuation of the MgO-NP fiber with respect to the imbalances of the interference. This term, labeled Δ*A*, has in general a smaller value than *A*, and we can write the effective SIR as:

(6) $$SIR=SIR_{network}-\Delta A=-10\log_{10}\left(N-1\right)+G-2\alpha S-\Delta A.$$

### 3.6. Quantification of the Quality of Detection

**Figure 3:**
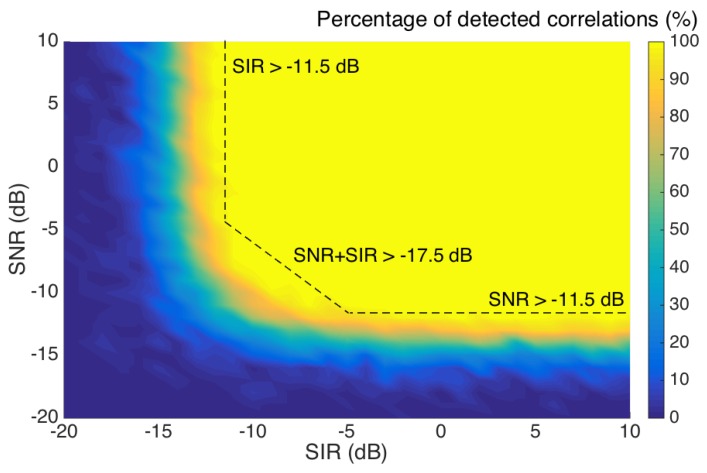
Percentage of correctly detected correlations, as a function of signal over interference ratio (SIR) and signal-to-noise ratio (SNR), by means of a Monte Carlo simulation applied on Rayleigh scattering signatures.

OBR detection is based on the cross-correlation between Rayleigh scattering signatures [13], which is known to be a noise-resilient operation when applied to spectral detection [14,27,28]. Due to the overlap of scattering signals, statistically independent on each other, and approximated as a white stochastic process [13,23], we need to quantify the limit of SNR and SIR at which the system is operating. A Monte Carlo simulation (M = 1000 order) has been performed by combining signatures at different SIR and SNR levels, and evaluating the percentage of detected correlations. The result is shown in Figure 3.

In order to account for 100% correct detection, with an operative margin, we can see the boundary limits for SIR and SNR. The system is limited by either SIR or SNR at the limit of −11.5 dB, with a transition region which can be approximated as a linear trend. In formulas, we can express the three conditions to be met in order to meet the OBR cross-correlation limits:

*SIR* > −11.5 dB (7)

*SNR* > −11.5 dB (8)

*SIR* + *SNR* > −17.5 dB (9)

where SIR and SNR are expressed in dB units.

### 3.7. Maximum Sensing Fiber Length

By replacing Equations (5–6) into Equations (7–9), and solving for the maximum value of *S*, we can obtain an estimate of the maximum length of each sensing region, for the MgO-NP fiber having (*G*, 2*α*) scattering characteristics. Hence, we can evaluate the maximum length of a sensing region *S_max_* as the minimum value of *S* that satisfies the SNR/SIR conditions:

(10) $$S_{max}=min\left\{\frac{11.5-10\log_{10}N+G-\Delta A}{2\alpha };\frac{11.5-10\log_{10}N+G-A+P_{smf}-P_{N}}{2\alpha };\frac{17.5-20\log_{10}N+2G-A-\Delta A+P_{smf}-P_{N}}{4\alpha }\right\}$$

using the approximation log_10_(*N* − 1) ≈ log_10_*N*, which is effective for high values of *N*.

The result in Equation (10) provides a closed-form expression for the upper bound of a SLMux system, i.e., the relationship between the maximum length achievable, *S_max_*, for a system with *N* channels, given a high-scattering fiber with gain *G* and losses 2*α*, taking into account the effect of impairments.

## 4. Performance Analysis

**Figure 4:**
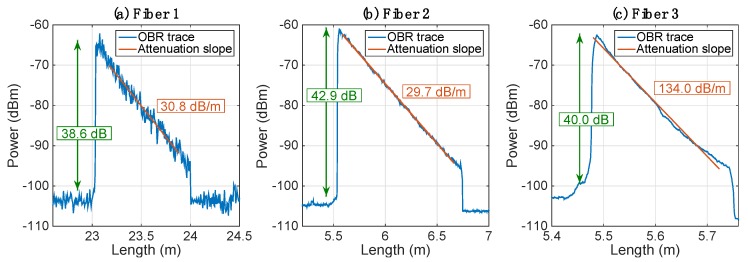
Scattering traces measured on the OBR instruments for three different MgO-NP fibers; data show the estimation of gain and two-way losses. (**a**) Fiber M01 used in [18]; (**b**) fiber M01 used in [19]; (**c**) fiber R04.

Several high-scattering fibers have been drawn, having different density and positioning of MgO-NP with respect to the fiber core. The scattering traces, measured with the OBR in different instances, show the estimate of the scattering parameters. Figure 4 shows the scattering traces of three different MgO-NP fibers, recorded on the OBR (using different gage length values). The trace appears very close to the sawtooth shape of Figure 2a, with an instantaneous rise of scattering level and a linear drop following the attenuation pattern. The first two fibers have similar gain and loss numbers, while the third fiber has similar gain, but much more significant losses.

Table 2 shows the parameters of all fibers used in the analysis.

Multiple fibers have been drawn, according to the methods described in [18,19,20,21,25], and the scattering parameters of seven fibers drawn with these methods, and reported on the OBR instrument, are reported in Table 2. Data report multiple fibers, drawn in different preforms that account for different location and distribution of elongated nanoparticles in the core.

The scattering gain recorded with the M01 preform, which appears as the most interesting method for SLMux, has a range of 5.7 dB (37.2–42.9 dB), while the two-way loss achieves a minimum of 22.1 dB/m and a maximum of 30.8 dB/m. This fiber has been used by Beisenova et al. and Molardi et al. [18,19,30], and the data are in agreement with Table 2, with some fluctuations also due to the random nature of nanoparticle distribution. Conversely, fibers drawn from the G22 fiber, achieve a higher gain (47.5–49.3 dB), but losses fall with a 10× higher rate, up to almost 300 dB/m [30]. In the middle between the two preforms, the R04 fiber pattern achieves a gain of 40.0 dB and losses of 134.0 dB/m.

**Figure 5:**
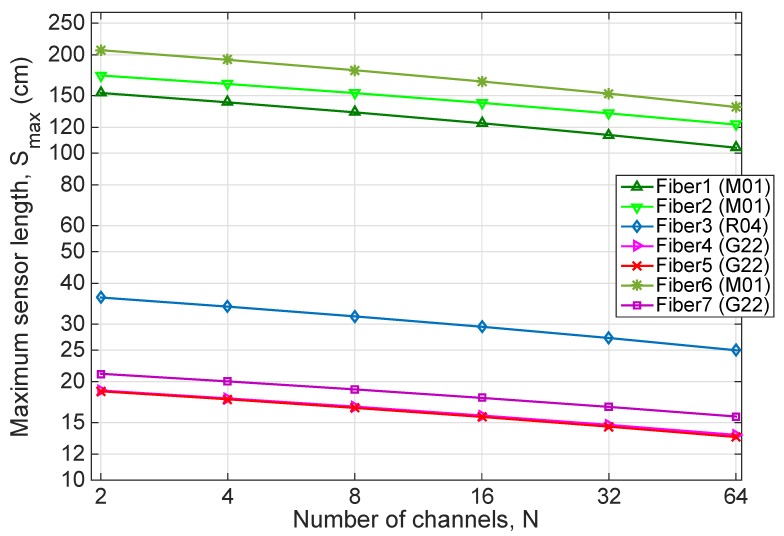
Maximum sensor length *S_max_*, as a function of number of channel *N*, in ideal conditions (*A* = 0 dB, Δ*A* = 0 dB), for the fibers listed in Table 2.

The performance of the seven fibers listed in Table 2, in ideal conditions (i.e., excess losses *A* = 0 dB, loss variation Δ*A* = 0 dB), is listed in Figure 5; in the chart, the maximum sensing length for the MgO-NP fibers is reported for each channel number. M01 fibers, having a smaller attenuation, have the best performance figure, due to the lower attenuation. The 6th fiber is the best performing method, achieving 206.7 cm of maximum sensing length in 2-channel configurations, down to 138.6 cm for 64 channels. The other M01 fibers have lower length, approximately 16% less for the 2nd fiber, and 26% lower for the 3rd fiber, with a similar slope. The R04 performance is lower; the maximum length is 36.2 cm for two channels, down to 25.0 cm for 64 channels. The G22 fiber type yields the lowest performance rating, where the maximum length achievable with this preform ranges from 18.7 cm to 21.1 cm for two channels, falling to 13.5–15.6 cm for the 64 channels.

The performances are dominated by the attenuation term: the increase of gain observed for G22 fibers does not suffice to cope with the higher losses, hence the performances of M01 fibers appear to always outperform other methods.

**Figure 6:**
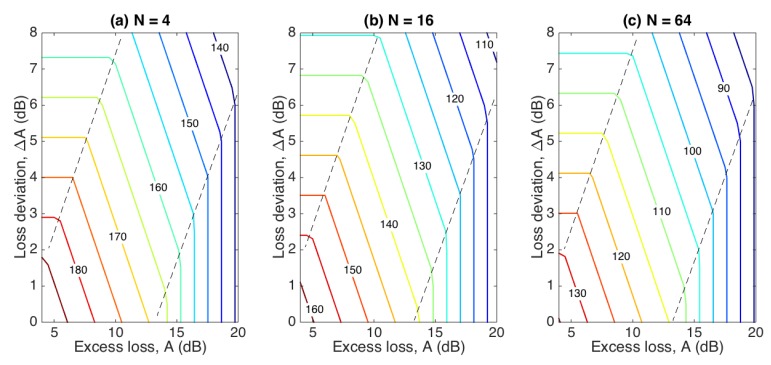
Maximum length *S_max_* of the sensing region (text, in centimeters) for the 6th fiber, M01 type, as a function of the impairments parameters *A* and Δ*A*, evaluated for (**a**) 4 channels; (**b**) 16 channels; (**c**) 64 channels.

The impact of impairments has to be assessed, for a system to take into account all effects. Considering, for simplicity, the 6th fiber as the most performing SLMux sensing fiber, we can evaluate the joint impact of impairments (*A*, Δ*A*), on the whole fiber length, evaluating the reduction of *S_max_* due to impairments. Results are shown in Figure 6, reporting the reduction of the maximum length as *A* and Δ*A* increase. We identify three regions: on the left and right parts of the curve, the maximum length is limited by Δ*A* and *A*, respectively, showing a saturation effect; in the inner region, the maximum length has a linear dependence on both impairments.

A well-designed SLMux, which operates to minimize the losses, minimizing the number of components, can count on the following excess losses, two-way (with numbers taken from component datasheets or splicer estimates): splice losses 0.05 dB ± 0.04 dB; connector losses 0.36 dB ± 0.08 dB for each pair of FC/APC connectors through a mating sleeve; excess losses of the splitter ranging from 0.9 dB to 6.6 dB; variation of splitting ratio per channel up to 15%, which corresponds to −0.15 dB to +0.15 dB. Hence, for a well-designed SLMux network, we can estimate *A* ~ 4.5 dB, and Δ*A* ~ 0.4 dB. In a worst-case scenario the imbalance was estimated as Δ*A* ~ 5 dB and a distribution network with multiple splitters was used, bringing the excess losses to about 11 dB [18]. In practical cases, among the several SLMux systems implemented by the authors, the Δ*A* parameter is the easiest to be controlled, since the user can assign the weakest port of the splitter to the N-th channel (longest), and the strongest signal to the first port, as it is more resilient to interferences. Conversely, splitter losses and excess losses are hard to be controlled. Empirically, we observe that *A* = 7 dB, and Δ*A* = 2 dB, are realistic parameters for the typical SLMux system. Hence, we can consider these three cases as benchmark: best case scenario *A* = 4.5 dB and Δ*A* = 0.4 dB; typical scenario *A* = 7 dB and Δ*A* = 2 dB; worst case scenario *A* = 11 dB and Δ*A* = 5 dB.

**Figure 7:**
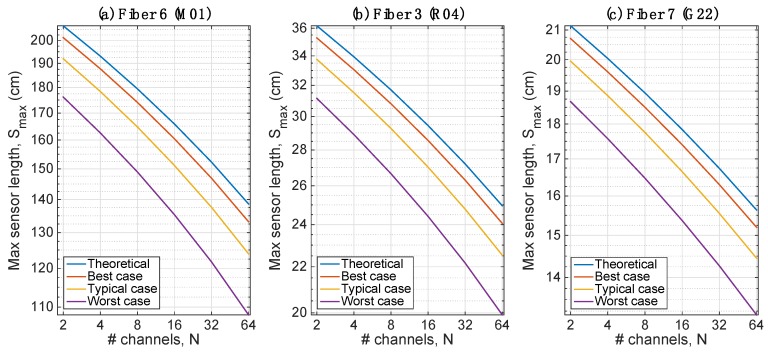
Maximum sensor length as a function of number of channels, accounting the impairments: (**a**) Fiber 6, M01; (**b**) Fiber 3, R04; (**c**) Fiber 7, G22.

Figure 7 evaluates the penalty due to impairments for fibers 6 (M01), 3 (R04) and 7 (G22). For the M01 fiber, we observe a penalty of 5.4 cm in the best case, 14.7 cm in the typical case and 30.5 cm in the worst case with respect to the ideal case. For the R04 fiber, the penalties are 0.9 cm, 2.4 cm and 5.0 cm, respectively. For the G22 fiber, the penalties are 0.4 cm, 1.2 cm and 2.5 cm respectively, about half of the R04 fiber. The chart shows that the inclusion of impairments does not alter the logarithmic dependence of the maximum sensing length with the number of channels, adding a penalty that can be quantified as a constant margin.

**Figure 8:**
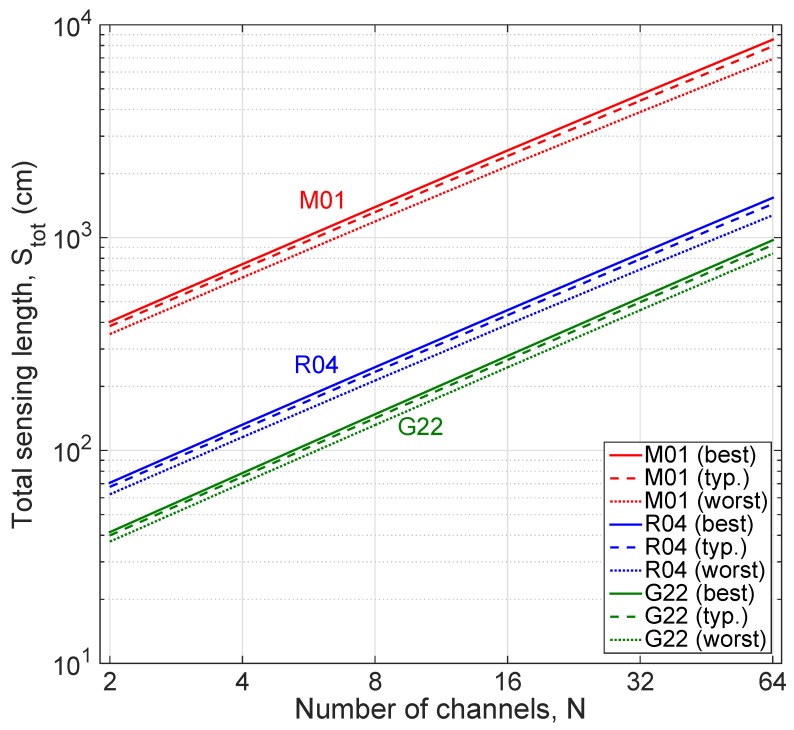
Total sensing length *S_tot_* for each fiber (fiber 6, M01; fiber 3, R04; fiber 7, G22), as a function of the number of SLMux channels.

The total sensing length that can be interrogated *S_tot_*, which corresponds to the sum of all the lengths associated to each channel *S_tot_ = NS_max_*, is shown in Figure 8. The M01 fiber, having the best performance rating for multiplexing, can achieve a sensing length of up to 85 m, using all the 64 channels, or up to 7.5 m for four channels, which is a length that covers a wide array of sensing applications. Even considering their length limitations, R04 and G22 fibers can achieve total sensing lengths of 132 cm and 78 cm, respectively, for four channels, and 15 m and 9 m for the maximum number of channels.

The results of the performance analysis are recapped in Table 3, reporting *S_max_* and *S_tot_* for all the MgO-NP fibers, in all the working conditions. We can observe the multiple trade-off existing between the maximum total active length of SLMux, and the length per sensing channel; the first element increases linearly with *N*, while the second term decreases logarithmically with *N*.

## 5. Range Extension through FBGs

As shown in by Yan et al. and Molardi et al., it is possible to efficiently inscribe FBGs on the MgO-NP fiber [24,30]. The effect of FBGs is to increase the overall reflectivity over the Rayleigh scattering floor, by adding the Bragg grating spectrum on top of the Rayleigh scattering. A correct application of FBG sensors is for range-extending the SLMux: at the tail of the MgO-NP fiber, we can continuously add FBGs (or, more consistently, a continuous FBG), in order to increase the baseline reflected trace.

The work reported by Molardi et al. provides a key estimate of the FBG-over-scattering performance, by reporting an FBG inscribed over a M01 fiber type, similar to the first fiber of Table 2 [30]. In this work, two FBGs have been inscribed, evaluating the enhancement of local reflectivity with respect to the MgO-NP fiber baseline scattering values: a weak grating, having 9.5 dB enhancement over the scattering gain, and a strong grating having 28.0 dB enhancement over the scattering gain; both gratings are almost free of insertion loss, since the in-line losses are significantly lower than the fiber loss. Limiting the analysis to the M01 fiber, which guarantees the inscription of similar gratings, and using the 9.5 dB (weak grating) and 28.0 dB (strong grating) enhancement factors, we can then adapt the SLMux performance analysis model to the FBG range-extension for each M01 fiber.

**Figure 9:**
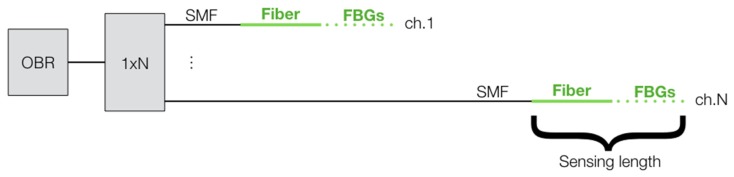
Sketch of the range-extended operation of SLMux through Fiber Bragg Gratings (FBGs).

The best working scenario is illustrated in Figure 9: at the location corresponding to *S_max_*, i.e., the maximum sensing length per channel, we can inscribe the gratings introducing a secondary gain, in accordance to the grating strength. Assuming that FBGs are lossless in such short length, where the FBG presence is showing no visible insertion loss, trivially the length of each sensor extension *S_FBG_* with the FBG is the solution of the conditions in Equation (10), replacing the gain with the grating enhancement factor *F* [30]:

(11) $$S_{FBG}=min\left\{\frac{11.5-10\log_{10}N+F-\Delta A}{2\alpha };\frac{11.5-10\log_{10}N+F-A+P_{smf}-P_{N}}{2\alpha };\frac{17.5-20\log_{10}N+2F-A-\Delta A+P_{smf}-P_{N}}{4\alpha }\right\}$$

and therefore, the maximum length, with extension, can be accounted by adding the original *S_max_* term to the *S_FBG_*:

(12) $$S_{extended}=min\left\{\frac{11.5-10\log_{10}N+G+F-\Delta A}{2\alpha };\frac{11.5-10\log_{10}N+G+F-A+P_{smf}-P_{N}}{2\alpha };\frac{17.5-20\log_{10}N+2G+2F-A-\Delta A+P_{smf}-P_{N}}{4\alpha }\right\}$$

**Figure 10:**
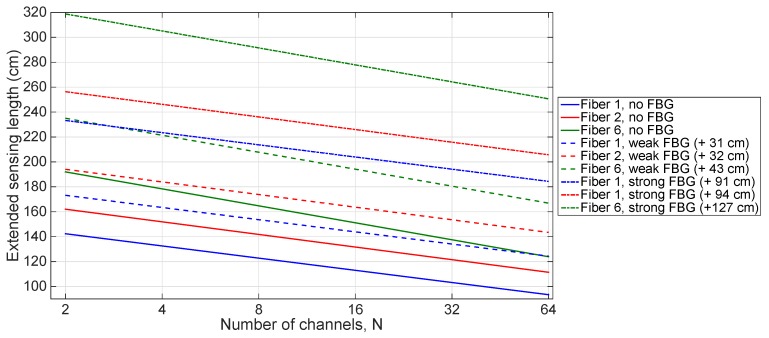
Extended sensing length (*S_extended_*) for M01 fibers 1, 2 and 6 in typical working conditions. The chart compares the SLMux performance in typical conditions, and when FBGs (weak, +9.5 dB; strong, +28.0 dB) are added to the span of sensing fiber.

Figure 10 shows the performance enhancement that can be obtained for range extension, in such conditions, with M01 fibers in typical conditions. Weak gratings provide a respective increase of 31, 32 and 43 cm for fibers 1, 2 and 6. Strong gratings, respectively, increase the limit of sensing length by 91, 92 and 127 cm, with a significant increase.

## 6. Discussion

The SLMux configuration finds its best application in biomedical applications, where the constraints on fiber arrangement for sensing are the strictest due to the requirement for invasiveness and bending radii. The work reported by Tosi et al. [4] reviews the main applications in biomedical engineering and medical devices that require distributed sensing with resolution below the centimeter level. The physical sensing capability of MgO-NP fibers interrogated via OBR allows detection of temperature and strain, and parameters derived from them such as the shape [21,22], which finds increasing attractiveness, and represents the current challenge. In other non-medical applications, it is easier to arrange the fiber into the configurations shown in Figure 1a, and real-time response is of less limited interest, hence a switch can be used as suggested in Figure 1b.

In light of these considerations, we can discuss the main findings obtained in this performance analysis, and understand their impact. At first, we observe that the type of fiber plays a fundamental role for the performance of the system. While Table 2 suggests that the attenuation and scattering gain are related, the attenuation term is the most important to determine the performance of the system: the fiber with the smallest attenuation is, under any operative condition, enabling the maximum sensing length. Hence, the use of M01 type of fibers is recommended, under this point of view.

The logarithmic dependence of the maximum sensor length with the number of channels has significant implications, since the maximum length of a 1 × 64 system is only 32% shorter than a 1 × 2, which means that a large sensing network performs similarly to a small number of channels in terms of accessible sensing length. This leaves the door open to the fabrication of medical devices that incorporate many fibers for multi-parameter sensing at the largest scale, without significant penalties. This is a key finding for shape or strain sensing in medical devices, which is inherently a multi-fiber, multi-point measurement [4] over several tens of centimeters.

The M01 fiber allows having sensing length well over one meter, while even the most attenuating fibers can achieve a range over 10 cm. This length is very attractive for medical applications, since this length, with multiple fibers, is needed to access some of the most advanced applications.

Thermal ablation has been considered by Beisenova et al. using up to four fibers [18]. In this application, the sensing length is up to ~5 cm, using a percutaneous device having length up to 20–25 cm. The data of performance analysis show the possibility of using a large amount of fibers, each with its length sufficient to cover both the thermally exposed part and the applicator length, avoiding splicing fibers too close to the sensing region.

Similarly, strain sensing requires 3–4 fibers (typically, three fibers for shape sensing, four fibers when temperature compensation is also accounted [22]) for each device under test. The epidural needle investigated in [21] requires interrogation over an 8 cm length, which is easily achieved by each fiber configuration. The analysis shows the feasibility, up to over 1 meter using M01 fibers, of using the MgO-NP fiber, not only for sensing, but also for covering the whole distance from the interrogator, leading out of the splitter.

Gastroscopy [31] and colonoscopy [32] devices have been developed, using a single fiber array, having length approaching the meter. A solid-state manometer for gastroscopy was reported by Arkwright et al. [31] using 32 FBGs over approximately 40 cm length, which covers the upper digestive apparatus length. SLMux shows the possibility of using the configuration for shape-sensing over a much longer length, to cover the whole apparatus from the entry point (nasal hole) to the bottom of the stomach. A colonoscopy device functionalized with FBGs was reported in [32], achieving a sensing length of 72 cm with 1 cm spacing. The authors concluded that the FBG spacing and spectral limitations control the catheter length, which is shorter than the colon length (~120 cm). The SLMux shows that at this length, several fibers can achieve sensing over 4–8 channels, which shows the feasibility of a shape-sensing colonoscope which can resolve the whole length. Virtually, the whole digestive apparatus (about 900 cm) can be covered by dedicating an 8–16 channel SLMux for each directional strain, according to the performance analysis. Similar considerations can be drawn for cardiovascular devices [22], which operate over few tens of centimeters of length. Clearly, such sensing systems can be arranged by proper geometrical design of the fibers around the medical apparatus; this analysis shows the feasibility of such architecture.

An important consideration is that the SLMux is achieved by means of several SMF spans used as spacers, which complicates the design. However, the SMF spans can be replaced by programmable delay lines [5], which can introduce an arbitrary delay over the fiber link, replacing a fiber time having the same in-line propagation delay.

The model hereby presented can be expanded for every scattering enhancement method, and for every OBR by using the adequate scattering parameters and the OBR noise floor values.

One consideration has to be drawn on the applicability of SLMux through MgO-NP fibers, with respect to other methods. The MgO-NP is, as a matter of fact, a fiber matching the core-cladding shape of the SMF, which can be spooled and drawn as a standard fiber through a repeatable fabrication process [20,25]. That means that, in the sensing fiber, the protective buffer is always present, making the fiber resistant to mechanical stress, bending and penetration in the tissues. Conversely, both UV exposure [17,23] and nanogratings [24] require stripping the fiber coating, which makes the fiber very susceptible of breaking, considering that the coating is missing for distances of several centimeters. Recoating does still not guarantee the same strength of the original jacket, and also increases the fiber thickness, which can be problematic in some applications. Such advantage is reduced when FBGs are inscribed [30], as what this process requires might need the removal of the fiber jacket, hence the portion of fiber gratings is more fragile.

Finally, the results frown in this analysis do not affect the spatial resolution of the OBR sensing methods, which is substantially dependent on the light source scanning grid, on the detector, and on the software implementation of the instrument. Also, the sensing accuracy is dominated by the spectral resolution of the OBR (8 pm in the model used in this work), while the power level in each location does not affect the correlation operator between signatures, as long as the SIR and SNR levels are over the threshold of Equations (7)–(9). As in Figure 3, the correlation operator is either working in all instances of detection, or is never reliable with a neat trend at the SIR/SNR threshold.

The performance analysis, furthermore, applies in principle to any OFDR system that cannot be wavelength-multiplexed; since the OBR uses broad-band fiber signatures, it cannot share the wavelength between multiple channels, and therefore is suitable for SLMux.

## 7. Conclusions

In conclusion, the performance analysis of scattering-level multiplexing in high-scattering distributed sensing has been reported, through an analytical model that takes into account the propagation of backscattered light and the noise and interference terms. Performance limits, for each fiber type, have been analytically identified for each type of MgO-NP fiber, and the results can be easily extended to any type of fiber having a high scattering content. The maximum sensing length has a logarithmic dependence on the number of channels, and sensing length can vary from tens of centimeters to over 1 meter, depending on the fiber type. FBGs can add reflectivity to the chain, and almost double the sensing length if needed. The results open the door to enable SLMux in biomedical devices, with sensing lengths adequate to address many medical applications.

## Figures and Tables

**Table 1:** List of the main parameters used in the performance analysis of SLMux.

Parameter	Label	Unit	Value
Scattering gain	G	dB	37.2–49.3 (Table 2)
Two-way fiber losses	2α	dB/m	22.1–298.0 (Table 2)
Number of SLMux channels	N	-	2–64
SMF backscattered power	P_SMF_	dBm	−102.7
OBR noise power level	P_N_	dBm	−110.7
Total interference power	P_INT_	dBm	= P_SMF_ + 10log_10_(N − 1) (max)
MgO-NP sensing fiber length	S	m	See Section 3.7
Maximum MgO-NP sensing length	S_max_	m	See Table 3
Total MgO-NP sensing length	S_tot_	m	= NS_max_
Extra two-way attenuations	A	dB	4.5–11
Maximum loss imbalance	ΔA	dB	0.4–5
Signal-to-noise ratio, ideal	SNR_network_	dB	See Section 3.4
Signal-to-noise ratio, real	SNR	dB	See Section 3.5
Signal-to-interference ratio, ideal	SIR_network_	dB	See Section 3.4
Signal-to- interference ratio, real	SIR	dB	See Section 3.5
Extra FBG power gain	F	dB	9.5–28.0
Maximum FBG chain length	S_FBG_	m	
Range-extended max. SLMux length	S_extended_	m	= S_max_ + S_FBG_

**Table 2:** Scattering parameters and fiber characteristics of MgO-NP specialty fibers.

Fiber ^1^	Reference	Preform Type ^2^	G [dB]	2α [dB]
1	Figure 4a [18]	M01	38.6	30.8
2	Figure 4b [19]	M01	42.9	29.7
3	Figure 4c	R04	40.0	134.0
4		G22	47.5	298.0
5	[29]	G22	46.1	292.0
6	[30]	M01	37.2	22.1
7		G22	49.3	273.3

^1^ Fibers are listed in chronological order of testing. ^2^ The preform type characterizes the label used by INPHYNI Institute to characterize the fibers.

**Table 3:** Recap of the performance analysis of SLMux; the table reports the maximum sensing length per channel (S_max_) and the total sensing length (S_tot_), for each fiber type in theoretical, best, typical and worst operative conditions for *N* ranging from 2 to 64 channels. Conditions: I = ideal; B = best case; T = typical case; W = worst case.

Fiber	Cond	S_max_ (cm)						S_tot_ (cm)					
		2	4	8	16	32	64	2	4	8	16	32	64
1 M01	I B T W	153 149 142 131	143 139 133 121	133 129 123 111	124 120 113 102	114 110 103 92	104 100 93 82	306 298 285 262	572 557 530 485	1067 1036 982 891	1977 1915 1808 1626	3641 3517 3304 2940	6657 6408 5982 5255
2 M01	I B T W	173 169 162 150	163 159 152 140	153 149 142 130	143 139 132 120	132 128 122 110	122 118 111 100	346 338 324 301	652 635 608 561	1222 1190 1135 1040	2282 2217 2107 1918	4240 4110 3889 3512	7830 7572 7130 6376
3 R04	I B T W	36 35 34 31	34 33 32 29	32 31 29 27	29 29 27 24	27 26 25 22	24 24 23 20	72 71 68 62	136 132 126 116	254 246 234 213	471 457 432 391	870 842 793 709	1597 1540 1442 1275
4 G22	I B T W	19 18 18 17	18 17 17 16	17 16 16 15	16 15 15 13	15 14 14 12	14 13 13 11	38 37 35 33	71 70 67 62	134 131 125 116	252 246 235 216	472 459 437 399	879 853 809 734
5 G22	I B T W	19 18 18 16	18 17 17 15	17 16 16 14	16 15 14 13	15 14 13 12	14 13 12 11	37 37 35 33	71 69 66 61	133 130 124 116	250 243 232 213	466 453 431 392	867 840 795 719
6 M01	I B T W	207 201 192 176	193 188 178 163	179 174 165 149	166 160 151 135	152 147 138 122	139 133 124 108	413 403 384 352	772 751 714 650	1436 1393 1318 1192	2654 2567 2419 2165	4872 4698 4402 3895	8873 8525 7931 6918
7 G22	I B T W	21 21 20 19	20 20 19 18	19 19 18 16	18 17 17 15	17 16 16 14	16 15 14 13	42 41 40 37	80 78 75 70	152 148 142 132	285 278 266 246	536 522 498 457	1001 973 925 843

**Figure A1:**
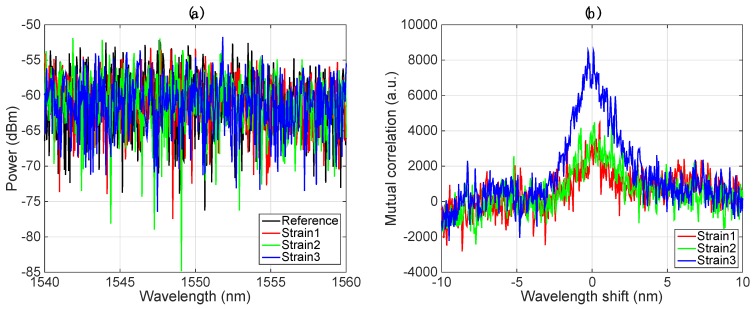
(**a**) Scattering traces of a MgO-NP fiber (reported, for visualization purposes, on the 1540–1560 nm range), in reference conditions and for 3 different values of strain. (**b**) Mutual correlation of the reference signature with the signatures acquired for the 3 strain values.

**Figure A2:**
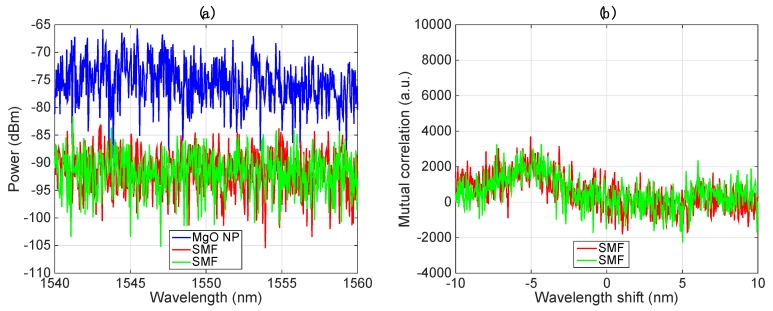
(**a**) Scattering trace of a MgO-NP fiber and 2 sections of SMF fibers, acquired in different location. (**b**) Mutual correlation between the MgO-NP and the 2 SMF fibers.

**Figure A3:**
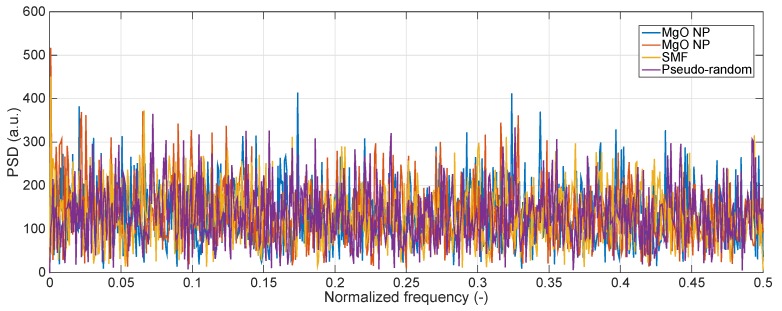
Power spectral density (PSD) or 2 MgO-NP and one SMF signatures, compared with a pseudo-random Gaussian process.

**Figure A4:**
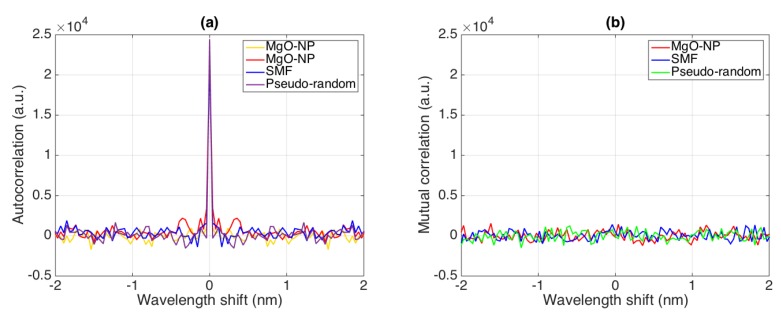
(**a**) Autocorrelation of scattering trace of 2 MgO-NO fibers, one SMF fiber, and a pseudo-random Gaussian process; (**b**) mutual correlation between the first MgO-NO signatures and the other MgO-NP, SMF, and pseudo-random processes.

## Appendix A

This Appendix A provides an experimental evidence of the assumptions relating the statistical dependency and variance of the Rayleigh scattering signatures acquired by the OBR at different locations [16], which is at the base of Sect. 3.3 and was initially reported in [19]. Here we aim to prove in a more complete way that: (1) a signature of a MgO-NP at the generic location is well correlated with the signature acquired at the same location in presence of a physical parameter change (i.e., strain or temperature change); (2) signatures acquired at different locations are statistically independent, regardless on whether the signature is on a SMF or MgO-NP fiber; (3) signatures representing the packscattered power, in dBm, can be treated as a random process with normal distribution. To prove these statements, we consider the acquisition of multiple signatures on both SMF and MgO-NP fibers, and show the respective correlations; in the following charts we limit to few signatures for visualization purpose, however the analysis has been repeated at different locations validating the concept. The results hereby acquired and shown have been estimated on over 20 signatures, obtaining consistent results.

In Figure A1 we prove that the MgO-NP fiber behaves, in terms of signature shift, as a standard SMF fiber: given a signature acquired in a location, and the same signatures with 3 different strain values, we can see that a correlation peak exists and stands out from the noise floor. Through signal processing, as expressed in [16,18], we can retrieve the wavelength shift as the peak of correlation, which appears similar to the correlation traces of [18,19] (correlation quality 0.89–0.99 [16]). Through this validation, the OBR model [13] maintains its working principle in MgO-NP fibers as in a SMF standard fiber.

The OBR system works since the SMF contributions overlapping to the MgO-NP fiber sections are stastically independent from each other. We prove it, in Figure A2, by acquiring two SMF traces and one MgO-NP at different locations. By calculating the mutual correlations, we notice that there is no clear correlation peak, as in Figure A2b, and the correlation appears as a flat function, similar to the cross-correlation of two white stochastic processes.

Finally, Figure A3 proves that the scattering signatures can be approximated as a white noise. To validate this concept, we estimate the power spectral density (PSD) of the scattering traces of 2 MgO-NP fibers and one SMF fiber, and we compare them with a pseudo-random Gaussian noise, by means of Fast Fourier Transform (FFT). We can see that the traces are similar, with a flat spectrum on the whole range of frequencies, and the only difference being a marginal low-frequency noise on the fibers. Hence, we can approximate the overlap of traces as the linear combination of two uncorrelated signals with different variance, completing the model of SLMux performance. This scenario is further verified in Figure A4, which shows the substantial analogy of fiber signatures with a Gaussian process: the first chart shows the autocorrelation of each signature represented in Figure A3, and as expected the autocorrelation is close to a delta function at the center of the spectrum. The second chart shows the mutual correlation between the first MgO-NP signature and the other waveforms, which shows a null spectrum. This behavior is in full analogy with a Gaussian random process, and proves the assumption of this work.

## References

[1] Othonos A., Kalli K. (1999). Fiber Bragg Gratings: Fundamentals and Applications in Telecommunications and Sensing.

[2] Kersey A.D., Davis M.A., Patrick H.J., LeBlanc M., Koo K.P., Askins C.G., Putnam M.A., Friebele E.J. (1997). Fiber grating sensors. J. Lightw. Technol..

[3] Lee B. (2003). Review of the present status of optical fiber sensors. Opt. Fiber Technol..

[4] Tosi D., Schena E., Molardi C., Korganbayev S. (2018). Fiber optic sensors for sub-centimeter spatially resolved measurements: Review and biomedical applications. Opt. Fiber Technol..

[5] Bao X., Chen L. (2012). Recent progress in distributed fiber optic sensors. Sensors.

[6] Dominguez-Lopez A., Soto M.A., Martin-Lopez S., Thevenaz L., Gonzalez-Herraez M. (2017). Resolving 1 million sensing points in an optimized differential time-domain Brillouin sensor. Opt. Lett..

[7] Mihailov S.J., Grobnic D., Smelser C.W., Lu P., Walker R.B., Ding H. (2011). Bragg grating inscription in various optical fibers with femtosecond infrared lasers and a phase mask. Opt. Mater. Express.

[8] Askins C.G., Putnam M.A., Williams G.M., Friebele E.J. (1994). Stepped-wavelength optical-fiber Bragg grating arrays fabricated in line on a draw tower. Opt. Lett..

[9] Gasulla I., Barrera D., Hervás J., Sales S. (2017). Spatial Division Multiplexed Microwave Signal processing by selective grating inscription in homogeneous multicore fibers. Sci. Rep..

[10] Oh S.T., Han W.T., Paek U.C., Chung Y. (2004). Discrimination of temperature and strain with a single FBG based on the birefringence effect. Opt. Express.

[11] Tosi D. (2016). Simultaneous detection of multiple fiber-optic Fabry–Perot interferometry sensors with cepstrum-division multiplexing. J. Lightw. Technol..

[12] Aitkulov A., Tosi D. (2019). Design of an All-POF-Fiber Smartphone Multichannel Breathing Sensor with Camera-Division Multiplexing. IEEE Sens. Lett..

[13] Froggatt M., Moore J. (1998). High-spatial-resolution distributed strain measurement in optical fiber with Rayleigh scatter. Appl. Opt..

[14] Soller B.J., Gifford D.K., Wolfe M.S., Froggatt M.E. (2005). High resolution optical frequency domain reflectometry for characterization of components and assemblies. Opt. Express.

[15] Macchi E.G., Tosi D., Braschi G., Gallati M., Cigada A., Lewis E. (2014). Optical fiber sensors-based temperature distribution measurement in ex vivo radiofrequency ablation with submillimeter resolution. J. Biomed. Opt..

[16] Luna Innovations Incorporated White Paper, Optical Backscatter Reflectometry (OBR)—Overview and Applications. https://lunainc.com/landing-page/download-now-obrwp/.

[17] Parent F., Loranger S., Mandal K.K., Iezzi V.L., Lapointe J., Boisvert J.S., Baiad M.D., Kadoury S., Kashyap R. (2017). Enhancement of accuracy in shape sensing of surgical needles using optical frequency domain reflectometry in optical fibers. Biomed. Opt. Express.

[18] Beisenova A., Issatayeva A., Sovetov S., Korganbayev S., Jelbuldina M., Ashikbayeva Z., Blanc W., Schena E., Sales S., Molardi C. (2019). Multi-fiber distributed thermal profiling of minimally invasive thermal ablation with scattering-level multiplexing in MgO-doped fibers. Biomed. Opt. Express.

[19] Beisenova A., Issatayeva A., Korganbayev S., Molardi C., Blanc W., Tosi D. (2019). Simultaneous distributed sensing on multiple MgO-doped high scattering fibers by means of scattering-level multiplexing. J. Lightw. Technol..

[20] Blanc W., Mauroy V., Nguyen L., Shivakiran Bhaktha B.N., Sebbah P., Pal B.P., Dussardier B. (2011). Fabrication of rare earth-doped transparent glass ceramic optical fibers by modified chemical vapor deposition. J. Am. Ceram. Soc..

[21] Beisenova A., Issatayeva A., Iordachita I., Blanc W., Molardi C., Tosi D. (2019). Distributed fiber optics 3D shape sensing by means of high scattering NP-doped fibers simultaneous spatial multiplexing. Opt. Express.

[22] Parent F., Gérard M., Monet F., Loranger S., Soulez G., Kashyap R., Kadoury S. (2018). Intra-Arterial Image Guidance With Optical Frequency Domain Reflectometry Shape Sensing. IEEE Trans. Med. Imaging.

[23] Loranger S., Gagné M., Lambin-Iezzi V., Kashyap R. (2015). Rayleigh scatter based order of magnitude increase in distributed temperature and strain sensing by simple UV exposure of optical fibre. Sci. Rep..

[24] Yan A., Huang S., Li S., Chen R., Ohodnicki P., Buric M., Lee S., Li M.J., Chen K.P. (2017). Distributed Optical Fiber Sensors with Ultrafast Laser Enhanced Rayleigh Backscattering Profiles for Real-Time Monitoring of Solid Oxide Fuel Cell Operations. Sci. Rep..

[25] Blanc W., Dussardier B. (2016). Formation and applications of nanoparticles in silica optical fibers. J. Opt..

[26] Blanc W., Martin I., François-Saint-Cyr H., Bidault X., Chaussedent S., Hombourger C., Lacomme S., Le Coustumer P., Neuville D., Larson D. (2019). Compositional Changes at the Early Stages of Nanoparticles Growth in Glasses. J. Phys. Chem. C.

[27] Reeves R.D., Patel B.M., Molnar C.J., Winefordner J.D. (1973). Application of correlation analysis for signal-to-noise enhancement in flame spectrometry. Use of correlation in determination of rhodium by atomic fluorescence. Anal. Chem..

[28] Gong J.M., MacAlpine J.M., Chan C.C., Jin W., Zhang M., Liao Y.B. (2002). A novel wavelength detection technique for fiber Bragg grating sensors. IEEE Photonics Technol. Lett..

[29] Korganbayev S., Shaimerdenova M., Ayupova T., Sypabekova M., Bekmurzayeva A., Blanc W., Molardi C., Tosi D. (2019). Refractive Index Sensor by Interrogation of Etched MgO Nanoparticle-Doped Optical Fiber Signature. IEEE Photonics Technol. Lett..

[30] Molardi C., Paixão T., Beisenova A., Min R., Antunes P., Marques C., Blanc W., Tosi D. (2019). Fiber Bragg Grating (FBG) Sensors in a High-Scattering Optical Fiber Doped with MgO Nanoparticles for Polarization-Dependent Temperature Sensing. Appl. Sci..

[31] Arkwright J.W., Blenman N.G., Underhill I.D., Maunder S.A., Szczesniak M.M., Dinning P.G., Cook I.J. (2009). In-vivo demonstration of a high resolution optical fiber manometry catheter for diagnosis of gastrointestinal motility disorders. Opt. Express.

[32] Arkwright J.W., Underhill I.D., Maunder S.A., Blenman N., Szczesniak M.M., Wiklendt L., Cook I.J., Lubowski D.Z., Dinning P.G. (2009). Design of a high-sensor count fibre optic manometry catheter for in-vivo colonic diagnostics. Opt. Express.

